# A revision of Poa subsection Aphanelytrum (Poaceae, Pooideae, Poaeae, Poinae); and a new species, *Poa auriculata*

**DOI:** 10.3897/phytokeys.63.8198

**Published:** 2016-06-06

**Authors:** Paul M. Peterson, Robert J. Soreng

**Affiliations:** 1 Department of Botany MRC-166, National Museum of Natural History, Smithsonian Institution, Washington, DC 20013-7012 USA

**Keywords:** Anatomy, Aphanelytrum, descriptions, Dioicopoa, Homalopoa, illustrations, key, morphology, taxonomy, Tovarochloa

## Abstract

In this study the peculiar Andean grass genus *Aphanelytrum*, with two species, is reduced to Poa subsect. Aphanelytrum **comb. & stat. nov.** A third species, *Festuca reclinata*, is assigned to the subsection, which shows states transitional between a more typical *Poa* and *Aphanelytrum*. Poa subgen. Poa supersect. Homalopoa sect. Dioicopoa subsect. Aphanelytrum **comb. & stat. nov.** is characterized in having stooling perennials with decumbent to spreading culm bases that continuously branch and often root at low to mid-culm nodes, glabrous spikelets with long rachillas 1.2–4.2 mm long, short glumes less than ½ the length of the florets, and lemmas with bifid apexes that are mucronate to short-awned. We provide for the three species taxonomic discussions, morphological and anatomical descriptions, keys, illustrations, and a list of specimens. Also, we provide two new names, *Poa hitchcockiana* **nom. nov.** and *Poa sanchez-vegae* **nom. nov.**, and one new combination, *Poa reclinata* **comb. nov.** A new species, *Poa auriculata* **sp. nov.** from Peru, not thought to be a member of Poa subsect. Aphanelytrum, is presented. It is the first in the genus with prominent auricles. In addition, we place *Poa apiculata* in Poa subgen. Poa supersect. Homalopoa sect. Dioicopoa subsect. Tovarochloa **comb. & stat. nov.**

## Abstract (es)

En este estudio el género peculiar de gramíneas andinas *Aphanelytrum*, representado por dos especias, ha sido reducido a Poa subsecc. Aphanelytrum **comb. & stat. nov.** Una tercera especie es incluida en la subsección, *Festuca reclinata*, esta presenta características transicionales entre las especies más típicas de *Poa* y *Aphanelytrum*. Poa subgen. Poa supersecc. Homalopoa secc. Dioicopoa subsecc. Aphanelytrum **comb. & stat. nov.** se caracteriza por incluir plantas perennes, stoloniferas, con los culmos decumbentes o extensos, continuamente ramificados, a menudo enraizados en los nudos basales hasta los intermedios, las espiguillas son glabras con rachillas largas de 1.2–4.2 mm de longitud, las glumas más cortas son de la ½ de longitud de los flósculos y las lemas con los ápices bífidos son mucronadas hasta breve-aristadas. Nosotros proveemos para las tres especias discusiones taxonómicas, descripciones morfológicas y anatómicas, claves, ilustraciones y la lista de las muestras usadas. Tambien, proveemos dos nombres nuevos - *Poa hitchcockiana* **nom. nov.** y *Poa sanchez-vegae* **nom. nov.**, y una combinación -*Poa reclinata* **comb. nov.** Una nueva especie - *Poa auriculata* **sp. nov.** de Perú es presentada. Esta es la especie primera del género con prominentes aurículas. En adición, transferimos *Poa apiculata* a Poa subgen. Poa supersecc. Homalopoa secc. Dioicopoa subsecc. Tovarochloa **comb. & stat. nov.**

## Introduction

The genus *Aphanelytrum* (Hack.) Hack., first named without a description by Sodiro (1889) with a single species, “*Aphanelytrum decumbens* Hack.” (ex Sodiro, nom. nud.) is based on a single collection he made from Ecuador (Chase 1916). Hackel (1897) formally described it as a subgenus of *Brachyelytrum* P. Beauv.; Brachyelytrum subgen. Aphanelytrum Hack., including a single new species *Brachyelytrum procumbens* Hack. Hackel (1902) subsequently recognized *Aphanelytrum* as a genus with the single species *Aphanelytrum procumbens* (Hack.) Hack. This species is found at mid- to high elevations (2000−4050 m) in humid to montane forests in the Andes of Bolivia, Colombia, Ecuador, and Peru (Hitchcock 1927; Clayton and Renvoize 1986; Jørgensen and Ulloa Ulloa 1994; Renvoize 1998; La Torre 2002; Soreng et al. 2003; Lægaard 2005; Sánchez Vega et al. 2007). *Aphanelytrum peruvianum* Sánchez Vega, P.M. Peterson, Soreng & Lægaard, a second species, was described recently (Sánchez Vega et al. 2007) from Cajamarca, Peru.

*Aphanelytrum procumbens* is a peculiar grass for having spikelets with minute and unveined glumes and two or three florets that are widely-spaced because of the long flexuous rachillas that are ½ to ¾ as long as the florets, the lemmas are keeled and 5-veined (Chase 1916; Hitchcock 1927; Nicora and Rúgolo de Agrasar 1987; Watson and Dallwitz 1992; Lægaard 2005). *Aphanelytrum peruvianum* differs from *Aphanelytrum procumbens* in having 1–3(–4)-veined glumes (1–2 mm long), narrow leaf blades (0.2–1.2 mm wide), shorter culms (14–24 cm long), shorter internodes (3–13 mm long), shorter 3- or 5-veined lemmas (2.2–3.5 mm long), and shorter anthers (2–2.9 mm long) [Sánchez Vega et al. 2007].

The placement and evolutionary relationships of *Aphanelytrum* have been controversial since its inception. It was originally placed in the subfamily “Festuceae” [Festucoideae], applied in the broad sense of Bentham (1881), Hackel (1887), and Hitchcock (1935). Hackel mistook the upper individual florets for single-flowered spikelets and placed *Aphanelytrum in Brachyelytrum* [*Brachyelytrum procumbens*] within tribe Agrostideae, subtribe Stipinae. Chase (1916) reinterpreted the spikelet morphology, and placed the genus between the subtribes Melicinae and Centothecinae, which at that time were considered adjacent subtribes of the subfamily Festucoideae, tribe Festuceae. After major realignments of the classification of the Poaceae (e.g. Clayton and Renvoize 1986), the genus was placed in a much more narrowly defined subfamily Pooideae (syn. Festucoideae), tribe Poeae (syn. Festuceae) near *Poa* L. (Clayton and Renvoize 1986). *Aphanelytrum* and *Poa* have very similar leaf anatomical characteristics, as well as multi-flowered, membranous spikelets with 5-veined, keeled lemmas, glabrous ovaries, and caryopses with an oval hilum (Clayton and Renvoize 1986; Clayton et al. 2006). In the most recent Poaceae classification (e.g. Soreng et al. 2015) *Aphanelytrum* is placed as a synonym of *Poa* in the subtribe Poinae, as suggested in studies by Gillespie et al. (2008), Soreng et al. (2007, 2015), Soreng and Peterson (2012), and Refulio Rodriguez et al. (2012).

Based on ITS sequences, Gillespie et al. (2008) found the monotypic *Tovarochloa* (*Tovarochloa peruviana* T.D. Macfarl. & P. But) to be a weakly supported sister of *Aphanelytrum procumbens* and *Aphanelytrum peruvianum*. In a plastid *trn-TLF* derived tree, Refulio Rodriguez et al. (2012) verified this result showing a strongly supported *Aphanelytrum procumbens* sister to *Tovarochloa*, and published a section in *Poa* for *Tovarochloa* (nom. inval.; later validated as Poa sect. Tovarochloa (T.D. Macfarl. & P. But) Molinari, see Molinari-Novoa 2015), but they left the genus *Aphanelytrum* unplaced within *Poa*. *Tovarochloa peruviana* is a small, delicate and diminutive annual species with 1-flowered spikelets that was previously linked to *Dissanthelium* Trin. (Clayton and Renvoize 1986; Macfarlane and But 1982; Tovar 1993), now both are included within *Poa* Gillespie et al. 2008; Refulio Rodriguez et al. 2012; Soreng et al. 2015). Giussani et al. (in press) estimated the time of divergence of *Tovarochloa* and *Aphanelytrum* at between 1.24 and 5.05 mya.

*Festuca reclinata* Swallen, known only from the Páramo del Almorzadero in the Cordillera Oriental of Colombia, has been linked to *Aphanelytrum* based on exhibiting similar morphologies (Stančík and Peterson 2007; Sánchez Vega et al. 2007). The growth habit, panicles, and spikelet characteristics of *Festuca reclinata* are strikingly similar to those found in *Aphanelytrum peruvianum* and *Aphanelytrum procumbens* (Sánchez Vega et al. 2007). All three species have weak, decumbent culms with intravaginal branching, narrow few-spikeleted panicles, spikelets with long flexuous rachillas, and small glumes. In comparison with *Festuca reclinata, Aphanelytrum peruvianum* has smaller lemmas (2.2−3.5 mm vs. 7−8.5 mm in *Festuca reclinata*), smaller anthers (2−2.9 mm vs. 3.5−3.8 mm), and shorter spikelets (5−7 mm vs. 10−13 mm) [Sánchez Vega et al. 2007]. The strongly keeled lemmas in *Festuca reclinata* do not agree with its placement in *Festuca* (Sánchez Vega et al. 2007). Also like *Aphanelytrum* and *Poa*, *Festuca reclinata* has fused sheath margins, terete rachillas (vs. dorsoventrally compressed in *Festuca*), and lacks the thickened annulate callus typical of *Festuca*. *Aphanelytrum procumbens* has a caryopsis with short elliptical hilum, less than 1/5 the grain in length (typical of *Poa* vs. linear and proportionally longer in *Festuca*), but caryopses have not been observed in *Aphanelytrum peruvianum* or *Festuca reclinata*.

The main goal of this study is to present a systematic revision Poa subsect. Aphanelytrum comb. & stat. nov. that includes three species. We make a new combination for *Festuca reclinata*, and provide new names for *Aphanelytrum procumbens* and *Aphanelytrum peruvianum*. In addition, we include a key to the species, complete descriptions, illustrations, distribution, specimens examined, and comments for these three species. Furthermore, we place *Poa apiculata* in Poa subsect. Tovarochloa comb. & stat. nov.

While reviewing Peruvian specimens of *Poa*, Robert J. Soreng (RJS) found a peculiar collection by John J. Wurdack (*Wurdack 1145*) from Departamento Amazonas, Provincia Chachapoyas located on the summit of Puma-urcu that is similar to *Poa scabrivaginata* Tovar and *Festuca reclinata* but differed from the forgoing, and all other *Poa* species, in having auriculate collars. We describe this as a new species of *Poa*, but do not include it as a member of Poa subsect. Aphanelytrum.

## Materials and methods

Herbarium specimens from the following 13 herbaria were examined: AAU, BC, COL, CPUN, K, LPB, MA, MO, QCNE, US, USM, USZ, and W (Thiers 2013).

For leaf anatomy, 5 mm long leaf blades were taken from dried herbarium specimens, rehydrated in boiling water, and fixed in FAA for 24 hours. They were transferred to 70% ethanol, followed by a water rinse and treated for three hours in 50% hydrofluoric acid (Martens and Uhl 1980). After being neutralized and washed in water the specimens were dehydrated in 2,2-dimethoxypropane (Postek and Tucker 1976) and embedded in Polyfin (Polysciences, Inc.) paraffin wax. Transverse serial sections were made at 4 μm, stained with buffered Toluidine Blue O (Sakai 1973) and mounted in Lipshaw’s synthetic mounting resin. Photomicrographic images were captured using a Zeiss Standard 16WL compound microscope equipped with a Retiga 1300i digital camera using ImagePro (MediaCybernetics).

## Taxonomic treatment

### Poa subsect. Aphanelytrum

Taxon classificationPlantaePoalesPoaceae

(Hack.) Soreng & P.M. Peterson comb. & stat. nov.

urn:lsid:ipni.org:names:77155363-1

Poa subsect. Aphanelytrum within Poa subg. Poa supersect. Homalopoa (Dumort.) Soreng & L.J. Gillespie sect. Dioicopoa E. Desv., see Gillespie et al. 2007

#### Basionym.

Brachyelytrum subg. Aphanelytrum Hack., Die Nat. Pflanzenfam., Nachträge zu Teil II, Abteilung 2. 42. 1897.

*Aphanelytrum* (Hack.) Hack., Oesterr. Bot. Z. 52: 12. 1902. *Aphanelytrum procumbens* (Hack.) Hack., Oesterr. Bot. Z. 52: 13, text f. 1902.

*Aphanelytrum* Hack. ex Sodiro, Anales Univ. Centr. Ecuador 3(25): 480. 1889, nom. nud.

#### Type species.

Based on *Brachyelytrum procumbens* Hack. ≡ *Aphanelytrum procumbens* (Hack.) Hack. ≡ *Poa hitchcockiana*.

#### Diagnosis.

Poa subsect. Aphanelytrum differs from most species of *Poa* in having stooling perennials with decumbent to spreading culm bases that continuously branch and often root at low to mid-culm nodes, glabrous spikelets with long rachillas 1.2–4.2 mm long, short glumes less than ½ the length of the florets, and lemmas with bifid apices that are mucronate to short-awned.

#### Description.

Stooling perennials with intravaginal innovations. Culms 14–80 (–100) cm tall, decumbent to spreading near base, culm bases that continuously branch and often root at low to mid-culm nodes. Leaf blades 3–14 cm long, 0.2–5 (–5.5) mm wide, flat to loosely involute; ligules 1–3 mm long, membranous. Panicles few-flowered with 5–22 spikelets. Spikelets 5–18 mm long, 2–4-flowered, membranous, glabrous, disarticulating above the glumes and between the florets; rachilla 1.2–4.2 mm long, terete in cross section, often prolonged above upper floret; callus glabrous; glumes 0.1–3.5 mm long, less than ½ the length of the florets, 0–3 (–4)-veined; lemmas 2.2–9.6 mm long, 3- or 5-veined, lanceolate or ovate, apex bifid, mucronate to short-awned, if awned up to 2 mm long; paleas 2–7 mm long, apex bifid; lodicules 2, glabrous; stamens 3, anthers 2–4.7 mm long; ovaries glabrous. Caryopses compressed laterally or unknown.

Three species of northern to central Andes of South America.

#### Key to the species of Poa subsection Aphanelytrum

**Table:** 

1	Glumes veinless, 0.1−0.5 (–0.7) mm long, minute or absent	***Poa hitchcockiana***
–	Glumes veined, 1−3.6 mm long, lower glumes 1 or 3-veined, upper glumes 3 or 4-veined	**2**
2	Spikelets 10–13 cm long, 4-flowered; lemmas 6.6–8 mm long; leaf blades 3–5 mm wide; paleas 4.6–5.2 mm long; culm internodes 14–80 mm long	***Poa reclinata***
–	Spikelets 5–7 mm long, 3-flowered; lemmas 2.2–3.5 mm long; leaf blades 0.2–1.2 mm wide; paleas 2–3.2 mm long; culm internodes 3−18 mm long	***Poa sanchez-vegae***

### Poa hitchcockiana

Taxon classificationPlantaePoalesPoaceae

Soreng & P.M. Peterson nom. nov.

urn:lsid:ipni.org:names:77155364-1

Figs 1 , 2D , 4C

Brachyelytrum procumbens Hack., Die Nat. Pflanzenfam., Nachträge zu Teil II, Abteilung 2. 42. 1897. Aphanelytrum procumbens (Hack.) Hack., Oesterr. Bot. Z. 52: 13, text f. 1902.
Aphanelytrum decumbens Hack. ex Sodiro, Anales Univ. Centr. Ecuador 3(25): 480. 1889, nom. nud.

#### Type.

ECUADOR. Crescit in silvis opacis regionis subandinis, 2000 m, Jul 1887, *L. Sodiro, s.n.* (holotype: W-19813 seen digitally!; isotype: US-865406 fragm. ex W!).

#### Description.

Straggling and stooling perennials with intravaginal innovations. Culms 30–80 (–100) cm tall, decumbent to erect, delicate, glabrous; nodes 3–8 (–14); internodes 2–14 cm long. Leaf sheaths ½ to 4/5 as long as the internodes, membranous to hyaline, often shiny, upper sheaths open ½ the length, keeled; ligules 1–2 mm long, membranous to hyaline, apex erose, often lacerate; blades 6–14 cm long (flag leaf usually 4–6 cm long), 1.5–4.2 (–5.5) mm wide, flat, thin, lax, linear. Panicles 5–18 (–22) cm long, 2–5 cm wide, few-flowered with 10–22 spikelets, oblong; branches flexuous, effuse and spreading, the lower branches capillary with 2–5 spikelets immediately branching below, the upper branches usually with 2 spikelets. Spikelets 8–18 mm long, 2- or 3-flowered (often appearing 1-flowered with disarticulation of upper florets), laterally compressed, greenish; disarticulation between the florets; all florets usually perfect; rachilla joints 1.5–4.2 mm long, often prolonged above upper floret; glumes 0.1–0.5 (–0.7) mm long, without veins, minute or absent, apex acute or irregularly lobed or toothed; lemmas 5–9.6 mm long, 5-veined, lanceolate, apex acuminate, mucronate or short-awned, the awn up to 2 mm long; paleas 4–7 mm long, 2-keeled, apex bifid; lodicules 0.8–1.1 mm long, lanceolate, membranous, glabrous; stamens 3; anthers 2.8–4.7 mm long, yellowish; ovaries glabrous with two styles and two stigmas. Caryopses 3.25–4.2 mm long, compressed laterally, glabrous, hilum short.

#### Leaf anatomy.

The transverse section leaf anatomy of *Poa hitchcockiana* is C_3_, XyMS+ with non-radiate, spongy chlorenchyma, without adaxial palisade cells. There is a single primary vascular bundle associated with the midrib and a sclerenchyma girder 2 or 3-cells thick on the abaxial surface (Fig. 2D). Lateral primary vascular bundles are widely spaced and also have a few abaxial sclerenchyma cells.

**Figure 1.**
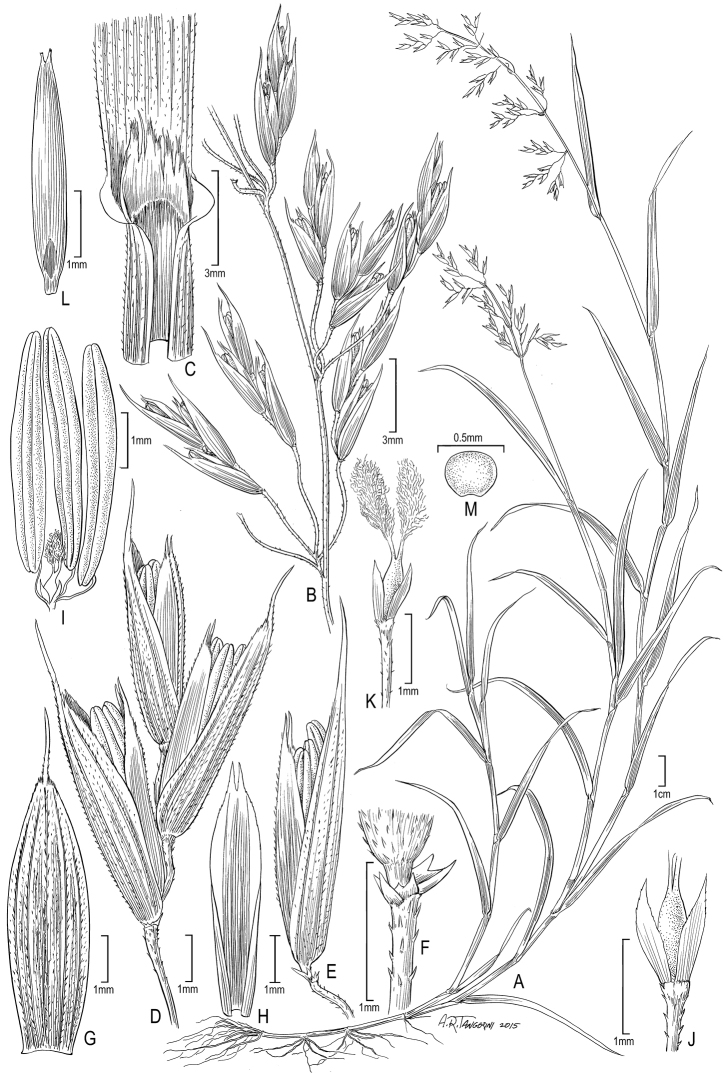
*Poa hitchcockiana*: **A** Habit **B** Panicle **C** Sheath, ligule, and blade **D** Spikelet **E** Lower floret with glumes at base **F** Glumes at base of lower floret **G** Lemma **H** Palea, ventral view **I** Stamens with ovary **J** Lodicules at base of ovary **K** Pistil, lodicules at base **L** Caryopsis **M** cross section of caryopsis. **A–D, J–K** (*Peterson 16571 & Refulio Rodriguez*) **E, F, L, M** (*Apollinaire 717 & Arthur*, US-913275).

**Figure 2.**
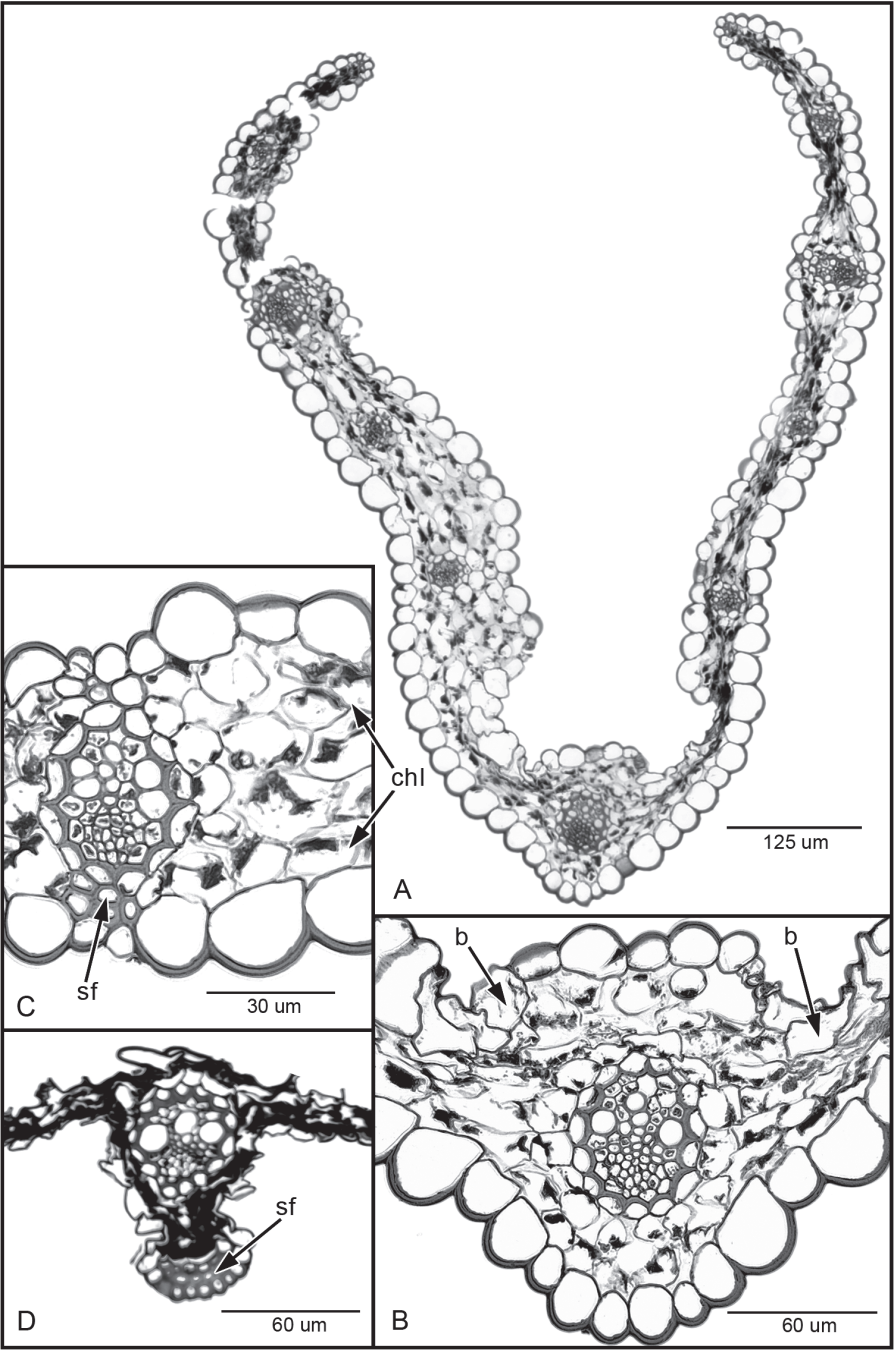
Leaf blade transverse sections of *Poa sanchez-vegae* (**A**–**C**) and *Poa hitchcockiana* (**D**). *Poa sanchez-vegae*: **A** Entire leaf blade **B** Midvein showing a single primary vascular bundle with bulliform cells (b indicated by arrows) **C** Lateral primary vascular bundle with a few associated abaxial sclerenchyma fibers (sf) and spongy chlorenchyma (chl). *Poa hitchcockiana*: **D** Midvein showing a single primary vascular bundle with abaxial sclerenchyma fibers (sf) and collapsed chlorenchyma.

#### Phenology.

Flowering year round [?], although no collections made in February, May, and September.

#### Distribution.

*Poa hitchcockiana* is found along the paramo or moist jalca vegetation between 2000–4025 m of the Cordillera de los Andes in Colombia, Ecuador, Bolivia, and Peru.

#### Conservation status.

Since the species is widespread it is of least concern (IUCN 2010). However, the typical size of populations is undocumented and it has been noted by the authors that the grass is sought after by grazers since it is often found growing among the protection of perennial shrubs.

#### Etymology.

Since the epithets *procumbens* and *decumbens* are blocked in *Poa* by the earlier *Poa procumbens* Curtis and *Poa decumbens* (L.) Scop., we provide a new name commemorating the “father of American agrostology,” Albert Spear Hitchcock (1865–1935).

#### Comments.

The only wide ranging species of Poa subsect. Aphanelytrum, *Poa hitchcockiana* also has the most unusual spikelet morphology with extremely long rachillas (1.5–4.2 mm long) and very short [0.1–0.5 (–0.7) mm long] to obscure or often absent, unveined glumes. *Poa* rachillas rarely exceed 1.5 mm, but some have spikelets with rachillas up to 2 mm long (e.g. Poa sect. Secundae V.L. Marsh ex Soreng) *Poa curtifolia* Scribn., *Poa hartzii* Gand., *Poa stenantha* Trin.; (Poa sect. Cenisiae Asch. & Graebn.) *Poa davisii* Bor; (Poa supersect. Homalopoa) *Poa bajaensis* Soreng, and a few other species in the *Homalopoa* (H) clade (see Soreng et al. 2010). Four species of the related genus *Nicoraepoa* Soreng & L.J. Gillespie have rachillas up to 3 mm long (Soreng and Gillespie 2007). However, there are no known species of *Poa* with unveined glumes.

#### Specimens examined.

**Bolivia. El Beni**: Bella Vista, 26 Dec 1923, *A.S. Hitchcock 22756* (US). **La Paz**: Murillo, 2450 m, 7 Apr 1981, *S.A. Renvoize 4269, T.A. Cope & S.G. Beck* (MO); 16°08'S, 68°07'W, 2900 m, 18 Mar 1987, *J.C. Solomon 16417* (MO, US); 16°10'S, 68°07'W, 3000 m, 1 Mar 1980, *J.C. Solomon 5240* (MO); 3100m, 16 Mar 1982, *T. Feuerer 10719B* (MO); 7 Apr 1989, *Feuerer 5855C* (MO). Santa Rosa, 3030 m, 4 Aug 1979, *S.G. Beck 1085* (US). Nor Yungus, 3300 m, *O. Buchtien 4268* (US); 3250 m, 3 Apr 1981, *S.A. Renvoize 4188 & T.A. Cope* (K, LPB); Franz Tamayo, 14°43'47"S, 69°04'17"W, 3998 m, 18 Jun 2005, *A.F. Fuentes 8338, R. Hurtado, I. Jiménez, E. Cuevas & R. Cuevas* (LPB, MO, USZ); Inquisivi, 16°48'00"S, 67°16'00"W, 3400–3500 m, 9 Mar 1991, *M. Lewis 38263* (MO). **Colombia. Boyacá**: Nevado de Cocuy, 3750 m, 10 Sep 1938, *J. Cuatrecasas 1360* (US); 4025 m, 7 Oct 1972, *A.M. Cleef & P.A. Florschultz 5960* (US). **Cauca**: 3700 m, 5 Apr 1985, *J.R.I. Wood 4784* (MO). **Cundinamarca**: Bogota, 10 Aug 1859, *A. Lindig 1009* (MO, US); Paramo de Chipaque, 3300 m, *R. Jaramillo M. 5340* (COL). **Meta**: Paramo de Sumapaz, 3700 m, *A.M. Cleef 7686* (COL). **Tolima**: 16 Dec 1984, *J.R.I. Wood 4650* (MO). Camino del Verjon, 3100 m, Jul 1911, *Apollinaire 717 & Arthur* (US-727001), *Apollinaire 717 & Arthur* (US-913275). J. *Celestino Mutis 5533* (MA, US). **Ecuador. Cañar**: Interandina, 3000 m, 2 Jul 1950, *M. Acosta Solís 16962* (US). **Carchi**: 00°49'00"N, 77°57'00"W, 3800 m, 10 Mar 1992, *S. Lægaard 101662* (AAU); 00°40'00"N, 77°52'00"W, 3400 m, 1 Nov 1993, *W.A. Palacios 11739* (MO, QCNE). **Imbabura**: 00°20'00"N, 78°00'00"W, 3600–3650 m, 7–8 Feb 1992, *S. Lægaard 101171* (AAU). **Loja**: Cajanuma, 04°05'S, 79°12'W, 2700–3100 m, 5 Mar 1987, *I. Grignon 84297* (AAU, MO, US). **Napo**: 00°56'00"S, 78°23'00"W, 3600 m, 16–18 Nov 1984, *S. Lægaard 53356* (AAU, QCA, US). **Pichincha**: Paso de Huanpango, 3280 m, Jul 1928, *G. Firmín 439* (US); Pedregal, 3400 m, 7 Jul 1944, M. Acosta Solís 8333 (US); Montes Pichinchas, 3700 m, 21 Jan 1856, *W. Jameson s.n.* (US), *Jameson 168* (US); Pasochoa near Quito, 1890, *L. Sodiro s.n.* (US); La Campiña, 3000 m, *T. Holmgren 649* (US). **Tungurahua**: Cordillera de Llanganates, 3000 m, 16 Nov 1939, E. Asplund 9737 (US). **Peru. Cusco**: Paucartambo, 2800 m, 17 Mar 2002, *P.M. Peterson 16571 & N.F. Refulio Rodriguez* (US, USM); Calca, 3430 m, 17 Mar 2002, *P.M. Peterson 16581 & N.F. Refulio Rodriguez* (US, USM); Pillco, 17 Apr 1967, *C. Vargas C. 19264* (US); Quispicanche, 13°35'32.8"S, 70°58'39.9"W, 3097 m, 20 Mar 2007, *P.M. Peterson 20582, R.J. Soreng & K. Romaschenko* (US, USM); **Moquegua**: El Abra, 2000 m, Mar 1967, *C. Vargas C. 19104* (US). **San Martín**: Huicungo, 7°58'S, 77°20'W, 2900–3150 m, 27 Jun 1999, *A. Cano s.n.* (SI, USM); *B. León 3797* (USM).

### Poa reclinata

Taxon classificationPlantaePoalesPoaceae

(Swallen) Soreng & P.M. Peterson comb. nov.

urn:lsid:ipni.org:names:77155361-1

Fig. 3

Festuca reclinata Swallen, Contr. U.S. Natl. Herb. 26(6): 254. 1949.

#### Type.

COLOMBIA. Departamento Santander, Cordillera Oriental, Paramo de Almorzadero, 3500–3700 m, 20 Jun 1940, *J. Cuatrecasas & H. Garcia Barriga 9970* (holotype: US-1798714!; isotypes: BC-635144 seen digitally!; COL-34839 seen digitally!).

#### Description.

Stooling perennials forming small tussocks with intravaginal innovations. Culms 30–40 cm tall, decumbent to erect, often weak, spreading to prostrate, culm bases continuously branch and often root at low to mid-culm nodes, glabrous; nodes 2 or 3 in distal half; internodes 1.4–8.0 cm long. Leaf sheaths about 2/3 as long as the internodes, membranous, greenish-white, scabrous, upper sheaths open 1/2 the length, collars flared; ligules 1–2.5 mm long, membranous, apex acute, ephemeral; blades 5–15 cm × 0.3–0.5 mm, flat, thin, lax, green, abaxially scabrous. Panicles 9–10 × 2–3 cm, few-flowered with 5–8 spikelets, flexuous, ovate, branched; branches glabrous. Spikelets 10–13 mm long, 4-flowered, glabrous; obovate; rachilla 1.2–2.4 mm long, minutely scabrous; glumes 1.3–3.6 mm long, membranous, lanceolate, green, glabrous, upper margins hairy; lower glumes 1.3–2 mm long, 1-nerved, apex acute; upper glumes 3–3.5 mm long, less than ½ as long as the florets, 3-veined, apex acuminate; lemmas 6.6–8 mm long, 5-veined, lanceolate, membranous, green, scabrous, apex bifid, two-dentate, awned between the teeth, the awn 1–2 mm long; paleas 4.6–5.2 mm long, membranous, keels scabrous, apex bifid; lodicules 0.6–0.8 mm long, lanceolate; anthers 2.7–3.3 mm long; ovaries glabrous. Caryopses not seen.

#### Leaf anatomy.

The leaf anatomy of *Poa reclinata* is C_3_, XyMS+ and the transverse sections have many widely spaced vascular bundles with small ribs; sclerenchyma is under both abaxial and adaxial epidermis, discontinuous, small, extending to the vascular bundles forming girders; bulliform cells are absent; epidermis is sparsely hairy. An anatomical description of *Poa reclinata* is also found in Watson and Dallwitz (1992) and Stančík and Peterson (2007, figs 13g, 77c–f).

#### Phenology.

Flowering in July.

#### Distribution.

*Poa reclinata* is known only from the type locality, the paramo of the Colombian Cordillera Oriental, Dept. Santander (Stančík and Peterson 2007).

#### Conservation status.

The species is rare and its conservation status is data deficient (IUCN 2010).

#### Etymology.

The specific epithet is probably in reference to the decumbent, spreading or prostrate culms, a frequent characteristic of the species in this subsection of *Poa*.

#### Comments.

Stančík and Peterson (2007) mentioned that the spikelets and panicles of *Festuca* [*Poa*] *reclinata* were similar to *Aphanelytrum procumbens* [*Poa hitchcockiana*], but the glumes in the former are veined. They provisionally placed *Festuca reclinata* in Festuca subg. Subulatae (Tzvelev) E.B. Alexeev sect. Glabricarpae E.B. Alexeev, as suggested by Aleexev (1986), along with *Festuca caldasii* (Kunth) Kunth and *Festuca woodii* Stančík. The latter two species of *Festuca* have open sheaths, flattened and hairy (hispid?) rachillas, and lemmas with rounded keels and annulate calluses, whereas *Poa reclinata* has fused sheaths ½ their length, terete rachillas that are minutely scabrous, and lemmas with compressed keels and smooth transitions from callus to lemma. We hope to confirm the placement of *Poa reclinata* in Poa subsect. Aphanelytrum in future DNA studies.

**Figure 3.**
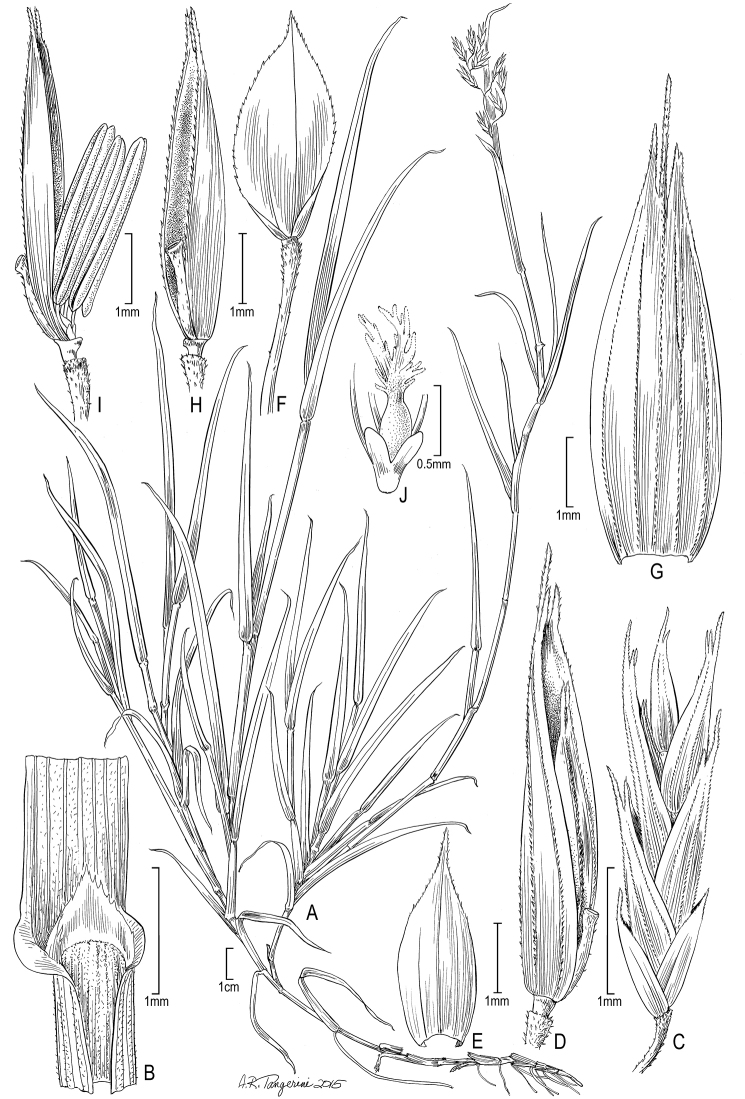
*Poa reclinata*: **A** Habit **B** Sheath, ligule, and blade **C** Spikelet **D** Lower floret **E** Upper glumes **F** Lower glume **G** Lemma **H** Palea and rachilla, lateral view **I** Stamens with palea **J** Lodicules and with pistil. (*Cuatrecasas 9970 & Garcua Barriga*, US-1798714).

### Poa sanchez-vegae

Taxon classificationPlantaePoalesPoaceae

Soreng & P.M. Peterson nom. nov.

urn:lsid:ipni.org:names:77155370-1

**Figure 4.**
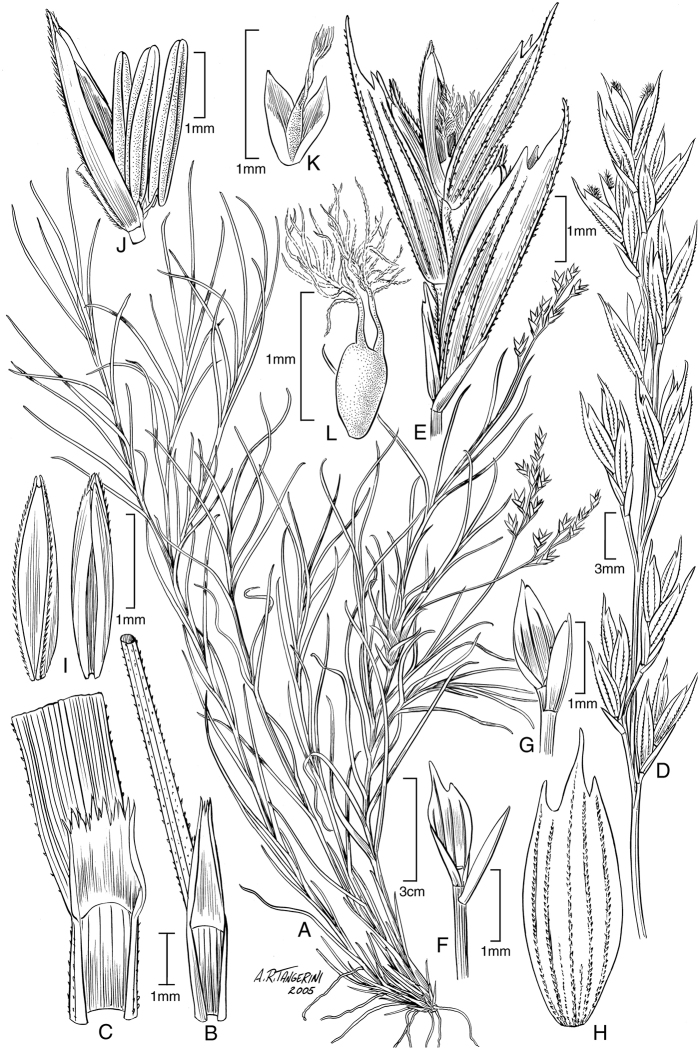
*Poa sanchez-vegae* (**A, B, D–L**) and *Poa hitchcockiana* (**C**). *Poa sanchez-vegae*: **A** Habit **B** Sheath, ligule, and blade **D** Panicle **E** Spikelet **F** Glumes, showing lower 1-veined and upper 4-veined **G** Glumes, showing lower 1-veined and upper 3-veined **H** Lemma **I** Paleas **J** Palea, anthers, and rachilla **K** Lodicules and pistil **L** Pistil. (*Sánchez Vega 11781, Sánchez Montoya, Cueva R. & Montoya*, US-3472470, US-3686568). *Poa hitchcockiana*: **C** Sheath, ligule, and blade (*Peterson 16571 & Refulio Rodriguez*).

Fig. 2A–C , 4A, B, D–L

Aphanelytrum peruvianum Sánchez Vega, P.M. Peterson, Soreng & Lægaard, J. Bot. Res. Inst. Texas 1(2): 842. 2007.

#### Type.

PERU. Departamento Cajamarca, Provincia Cajamarca, Distrito Cajamarca, Cerro Akumullca, al SO de Cajamarca, sobre la cima de la ladera occidental del Valle de Cajamarca (7°14'15"S Lat, 78°29'24"W Long), 3300 m, 20 Mar 2003, *I. Sánchez-Vega 11781, M. Sánchez-Montoya, R. Cueva R. & J. Montoya* (holotype: CPUN!; isotypes: AAU!, F!, HAO!, HUT!, LOJA!, MICH!, MO!, SI!, US-3472470!, US-3686568!, USM!).

#### Description.

Caespitose perennials. Culms 14−24 cm tall, with many culms near base, primary and secondary culms appressed, somewhat decumbent near base with intravaginal branching, culm bases continuously branch and often root at low to mid-culm nodes; internodes 3−18 mm long, numerous. Leaf sheaths longer than the internodes, membranous to hyaline, open to near base to open completely to base, slightly keeled; ligules 2−3 mm long, membranous to hyaline, decurrent, apex erose often lacerate; blades 3−7 cm long (flag leaf ca 1.6 mm long), 0.2−1.2 mm wide, flat to loosely involute, thin, linear, apex naviculate. Panicles 1.7−2.5 cm long, few-flowered with 5−10 spikelets; branches flexuous, the lower branches with two spikelets, the upper branches with single spikelet. Spikelets 5−7 mm long, usually 3-flowered, purplish, glabrous, disarticulating above the glumes and between the florets; lower and middle florets usually staminate; upper florets usually pistillate; rachilla joints 1.2−2 mm long, prolonged above the upper floret; glumes 1−2 mm long, subequal, apex acute, often mucronate; lower glume linear, 1-veined; upper glume oblanceolate, 3(4-)-veined, often toothed or irregularly lobed minutely bifid; lemmas 2.2−3.5 mm long, 3- or 5-veined, ovate, apex mucronate with two acute lobes on each side of the mucro, the mucro 0.1−0.3 mm long; paleas 2−3.2 mm long, 2-keeled, apex bifid; lodicules 0.7−0.8 mm long, lanceolate, membranous, glabrous; stamens 3; anthers 2−2.9 mm long, yellowish to purplish; ovaries glabrous with two styles and two stigmas. Caryopses glabrous.

#### Leaf anatomy.

The transverse section leaf anatomy of *Poa sanchez-vegae* is C_3_, XyMS+ with non-radiate, spongy chlorenchyma, without adaxial palisade cells (Fig. 2A–C). There are bulliform cells on the adaxial surface on either side of the midveins primary vascular bundle without additional sclerenchyma (Fig. 2B). However, there are a few abaxial sclerenchyma cells associated with the lateral primary vascular bundles (Fig. 2C).

#### Phenology.

Flowering in March.

#### Distribution.

*Poa sánchez-vegae* is known only from the type locality near the western highlands of the Cajamarca Valley and is found on rocky sites associated with jalca vegetation (humid alpine grass ecosystems) at 3300 m (Sánchez-Vega et al. 2007).

#### Conservation status.

*Poa sanchez-vegae* is rare and the conservation status is data deficient (IUCN 2010). However, the authors unsuccessfully searched for additional material of this species at the type locality on 26 Mar 2008, accompanied by two of the original collectors, Isidoro Sánchez-Vega and Juan Montoya. The site had been turned into a pine plantation.

#### Etymology.

Since the existing specific epithet was occupied in *Poa* we provide a new name commemorating Isidoro Sánchez Vega, a renowned Peruvian Botanist.

#### Comments.

Based on morphological characters, Isidoro Sánchez-Vega in consultation with Simon Lægaard, first identified the type collection of *Poa sanchesz-vegae* as an unknown species of *Aphanelytrum*. In Sánchez Vega et al. (2007), we described this taxon as a new *Aphanelytrum*, and mention that *Festuca reclinata* superficially resembled the genus. In addition, *Poa sanchez-vegae* aligned near or on an unusually long branch within *Poa* in preliminary cpDNA and ITS sequence analyses (Gillespie et al. 2007). Gillespie et al. (2008) included three accessions of *Poa hitchockiana* (≡ *Aphanelytrum procumbens*) that formed a clade sister to *Poa sanchez-vegae* (≡ *Aphanelytrum peruvianum*) which together was sister to two accessions of *Poa apiculata* (≡ *Tovarochloa peruviana*). These results clearly support our classification of *Poa sanchez-vegae* and *Poa hitchcockiana* as members of Poa subsect. Aphanelytrum.

### Other novelties

#### Poa subsect. Tovarochloa

Taxon classificationPlantaePoalesPoaceae

(Macfarl. & P. But) Soreng & P.M. Peterson comb. & stat. nov.

urn:lsid:ipni.org:names:77155398-1

Poa subsect. Tovarochloa within Poa subg. Poa supersect. Homalopoa (Dumort.) Soreng & L.J. Gillespie sect. Dioicopoa E. Desv., see Gillespie et al. 2007

##### Basionym.

*Tovarochloa* T.D. Macfarl. & P. But, Brittonia 34(4): 478. 1982.

##### Type species.

Based on *Tovarochloa peruviana* T.D. Macfarl. & P. But ≡ *Poa apiculata* Refulio.

##### Comments.

For consistency in rank, since *Poa apiculata* Refulio (≡ *Tovarochloa peruviana*) apparently is sister to the three species in Poa subsect. Aphanelytrum, we erect Poa subsect. Tovarochloa.

#### Poa auriculata

Taxon classificationPlantaePoalesPoaceae

Soreng & P.M. Peterson sp. nov.

urn:lsid:ipni.org:names:77155360-1

Fig. 5

##### Type.

PERÚ. Departamento Amazonas, Provincia Chachapoyas, summit of Puma-urcu southeast of Chachapoyas, occasional on dry cliff face, 3100–3200 m, 3 Jul 1962, *J.J. Wurdack 1145* (holotype: US-2382274!).

##### Diagnosis.

*Poa auriculata* differs from *Poa scabrivaginata* Tovar in having 4–6-flowered spikelets, a glabrous callus, glumes 2–3 mm long, and lemmas 2.5–3.8 mm long.

##### Description.

Caespitose, annuals or short-lived perennials. Culms 40–72 cm tall, erect, scabrous, shiny, often weak; nodes 3–5. Leaf sheaths 2/3 to 4/5 as long the internodes, membranous, greenish to stramineous, scabrous, upper sheaths open for 1/3 the length, keeled, summit with prominent triangular auricles; ligules 3.3–5 mm long, membranous to hyaline, apex erose, often split down the center; blades 6–15 cm long (flag leaf 3–6 cm long), 3–6 mm wide, flat, thin, lax, linear, scabrous. Panicles 5–11 cm long, 2.5–5 cm wide, ovate, open; branches flexuous, effuse and spreading with numerous spikelets, scabrous, the lower branches capillary. Spikelets 5–7 mm long, 4–6-flowered, glabrous, ovate, greenish-yellow tinged with purple; rachilla 0.4–1.0 mm long; glumes 2–3 mm long, membranous, subequal; lower glumes 2–2.5 mm long, 1-veined, linear lanceolate, apex acuminate; upper glumes 2.4–3 mm long, 3-veined, the veins not conspicuous, lanceolate, apex acute; lemmas 2.5–3.8 mm long, 5-veined, lanceolate, membranous; apex acute, unawned; paleas 2.3–3.7 mm long, membranous, 2-keeled, the keels scabrous, apex minutely bifid; lodicules 0.4–0.5 mm long, ovate, membranous, glabrous; stamens 3; anthers 1.9–2.1 mm long, yellowish; ovaries glabrous with two styles and two stigmas. Caryopses not seen.

##### Phenology.

Flowering in June and July.

##### Distribution.

*Poa auriculata* is known only from the type locality in Cordillera de los Andes of Peru near Chachapoyas between 3100–3200 m growing on a dry cliff face.

##### Conservation status.

The species is rare, but its conservation status is data deficient (IUCN 2010).

##### Etymology.

The specific epithet refers to the triangular auricles that are found on the summit of the sheaths, a feature that is unique among species of *Poa*.

##### Comments.

Initially RJS considered *Poa auriculata* to be related to species in Poa subsect. Aphanelytrum. We do not place the new species in Poa subsect. Aphanelytrum because the spikelets have short rachillas and are 4–6-flowered, the habit is annual to short-lived perennials with erect culms, and the lemmas are unawned without mucros. However, aside from the auricles, *Poa auriculata* is morphologically consistent with the 300 or so species that reside within Poa subg. Poa supersect. Homalopoa. We hope to include a sample of this species in upcoming molecular analyses.

*Poa scabrivaginata* differs from *Poa auriculata* in having 2-flowered spikelets (4–6 in *Poa auriculata*), a few cobwebby hairs on the callus (verses glabrous), glumes 3.6–4.3 mm long (verses 2–3 mm), and lemmas 4.2–4.5 mm long (verses 2.5–3.8 mm) [Tovar 1965]. *Poa aequatoriensis* Hack., another species possible to confuse with *Poa auriculata*, differs in having 2- or 3-, rarely 4-flowered spikelets, a few cobwebby hairs on the callus, and lemmas 3.6–4 mm long (Tovar 1965). *Poa aequatoriensis* is more wide ranging and has been reported in Colombia, Ecuador, and Peru whereas *Poa scabrivaginata* is known from the type (Depto. de Huánuco, Tambo de Vaca, *J.F. Macbride 4354* at US) and one other possible collection (Depto. de Cajamarca, Celendin, I. Sánchez Vega 2668 at MO) [Sylvester et al. in press].

**Figure 5.**
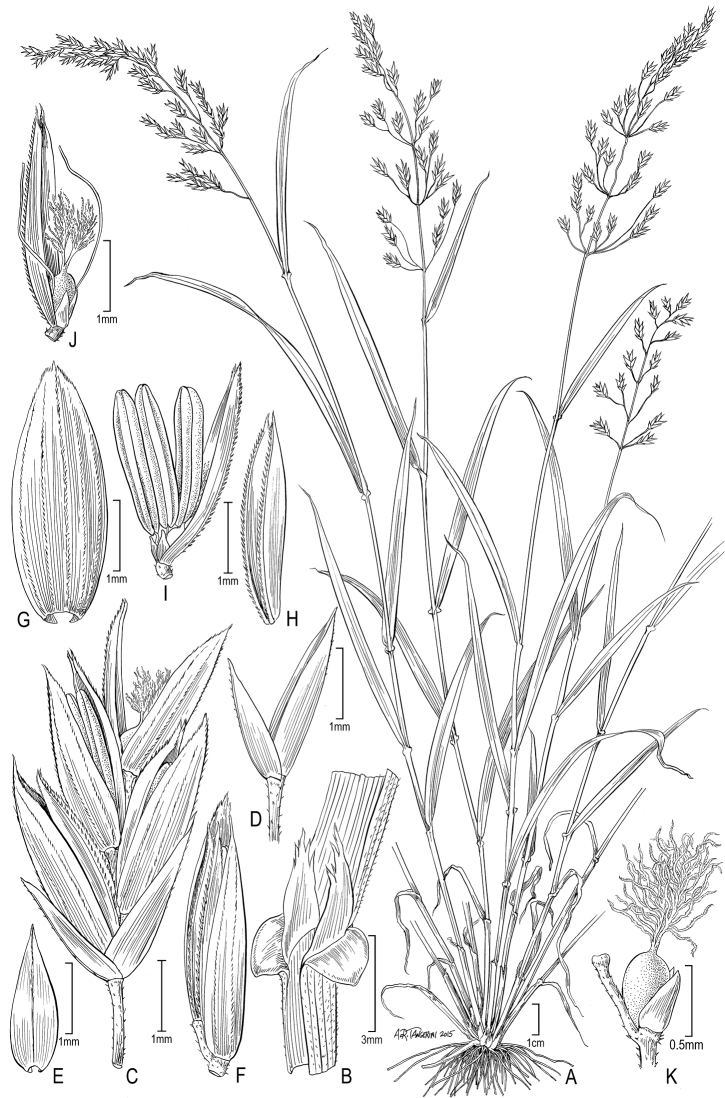
*Poa auriculata*: **A** Habit **B** Sheath, auricles, ligule, and blade **C** Spikelet **D** Glumes **E** Lower glume **F** Floret **G** Lemma **H** Palea, lateral view **I** Stamens with palea **J** Pistil enclosed in palea **K** Lodicules, pistil, and rachilla. (*Wurdack 1145*, US-2382274)

## Supplementary Material

XML Treatment for Poa subsect. Aphanelytrum

XML Treatment for Poa hitchcockiana

XML Treatment for Poa reclinata

XML Treatment for Poa sanchez-vegae

XML Treatment for Poa subsect. Tovarochloa

XML Treatment for Poa auriculata
